# A minimal model of peptide binding predicts ensemble properties of serum antibodies

**DOI:** 10.1186/1471-2164-13-79

**Published:** 2012-02-21

**Authors:** Victor Greiff, Henning Redestig, Juliane Lück, Nicole Bruni, Atijeh Valai, Susanne Hartmann, Sebastian Rausch, Johannes Schuchhardt, Michal Or-Guil

**Affiliations:** 1 Systems Immunology Lab, Department of Biology, Humboldt University Berlin, and Research Center ImmunoSciences, Charité University Medicine Berlin, Berlin, Germany; 2 Bayer CropScience N.V., Technologiepark, 38, 9052 Zwijnaarde, Gent, Belgium; 3 Studienmethodik und Statistik, Universitätsspital Basel, Basel, Switzerland; 4 Department of Molecular Parasitology, Humboldt University Berlin, Berlin, Germany; 5 MicroDiscovery GmbH, Berlin, Germany; 6 Contributed equally to this study

## Abstract

**Background**

The importance of peptide microarrays as a tool for serological diagnostics has strongly increased over the last decade. However, interpretation of the binding signals is still hampered by our limited understanding of the technology. This is in particular true for arrays probed with antibody mixtures of unknown complexity, such as sera. To gain insight into how signals depend on peptide amino acid sequences, we probed random-sequence peptide microarrays with sera of healthy and infected mice. We analyzed the resulting antibody binding profiles with regression methods and formulated a minimal model to explain our findings.

**Results**

Multivariate regression analysis relating peptide sequence to measured signals led to the definition of amino acid-associated weights. Although these weights do not contain information on amino acid position, they predict up to 40-50% of the binding profiles' variation. Mathematical modeling shows that this position-independent ansatz is only adequate for highly diverse random antibody mixtures which are not dominated by a few antibodies. Experimental results suggest that sera from healthy individuals correspond to that case, in contrast to sera of infected ones.

**Conclusions**

Our results indicate that position-independent amino acid-associated weights predict linear epitope binding of antibody mixtures only if the mixture is random, highly diverse, and contains no dominant antibodies. The discovered ensemble property is an important step towards an understanding of peptide-array serum-antibody binding profiles. It has implications for both serological diagnostics and B cell epitope mapping.

## Background

The functional antibody repertoire (FABR), the set of all antibodies produced by plasma cells at any one time, determines the immune system's perception of the antigen universe. The FABR is shaped throughout the life of an individual by various stages and selection events during B cell development that take place in the fetal liver, in the bone marrow and in secondary lymphatic organs. As the FABR is subject to constant change due to continuous antigen encounter and establishment of immunological memory [1], it encompasses a variety of specificities and affinities for a wide range of antigens [2]. The FABR's investigation thus provides the possibility to gather information about both past and on-going immune responses, and ultimately about the immune state of the body [3].

Since the FABR is highly diverse and the production of antibodies is a hallmark of many infectious and autoimmune diseases, high-throughput immunoblot and microarray technologies have been used intensively for large-scale profiling of serum antibody binding [4-9]. Antibody profiling data is widely used for serological diagnostics by exploiting the fact that sera of control and diseased individuals may differ substantially in their FABRs [7,8,10-12]. Currently, serum-antibody profiling is usually performed by incubating a serum sample with a peptide or protein microarray. Afterwards, the reactivity of antibodies is estimated by measuring the fluorescence from a fluorochrome-coupled secondary antibody that binds to the constant region of the subset of serum antibodies studied [13,14].

The importance of peptide microarrays as a tool for serological diagnostics has strongly increased over the last decade. However, interpretation of the binding signals is still hampered by our limited understanding of the technology [15]. This is in particular true for arrays probed with antibody mixtures of unknown complexity, such as sera. To gain insight into how signals depend on peptide amino acid sequences, we probed random-sequence peptide microarrays with sera of healthy and infected mice.

For prediction of antibody binding profiles, we use a multivariate regression model based exclusively on the peptide library's amino acid composition without taking into account amino acid positional information. This approach is related to methods of linear B cell epitope prediction which rely on propensity scales for epitope prediction [16-19]. Our method contrasts, however, with previously reported quantitative structure-activity relationship (QSAR) modeling which, in conjunction with physico-chemical properties, relates amino acid positions *and *amino acid compositions of peptides and monoclonal antibodies to various response variables [20-22]. We propose to examine, in vitro and in silico, the extent to which the validity of our approach depends on the composition of antibody mixtures.

The regression model led to the definition of amino acid-associated weights (AAWS) as predictors of antibody-peptide reactivity. We found that the position-independent peptide amino acid composition accounts for up to 40-50% in variation of antibody-peptide binding for healthy mice.

We demonstrate with a mathematical model the ensemble properties of highly diverse, random antibody mixtures in which no antibody dominates. We call these mixtures "unbiased" and show that the properties of unbiased mixtures are the foundation to a high predictive performance of AAWS. We hypothesize that serum antibodies of healthy individuals resemble an unbiased mixture, while during an acute immune response, specific antibodies dominate antibody-peptide binding thus lowering predictive performance. Based on in silico and in vitro evidence, our work thus suggests that the faithfulness of antibody-peptide binding prediction with propensity scales [16-19] decreases with increasing antibody dominance in a mixture.

## Results

In order to investigate the binding of antibody mixtures to large random-sequence peptide libraries, we asked two main questions: i) what is the impact of the peptides' amino acid composition on the binding to serum antibodies, ii) and how does the serum-antibody composition influence binding prediction?

### Experimental setup

To study the impact of amino acid composition of random-sequence peptide libraries on measured signal intensity, serum samples from 15 BALB/c mice bred under specific pathogen-free (SPF) conditions were collected. These mice were infected with HB (Additional file 1, Figure S1). Further serum samples were collected at 10 dpi (days post infection; 15 samples), at 14 dpi (13 samples) and at 18 dpi (15 samples) totaling 58 serum samples. Microarrays of *n*_Pep _= 255 random-sequence peptide probes (hereafter referred to as standard library) were incubated with the serum samples. The peptide arrays used have been shown to be suitable for serological diagnostics by Bongartz *et al. *[10]. Each probe consisted of *l *= 14 out of 20 proteinogenic amino acids. IgM and IgG antibody binding was simultaneously detected by means of isotype specific fluorochrome-labeled polyclonal secondary antibodies. In addition to serum samples, the peptide library was incubated separately with 13 different human monoclonal IgG antibodies.

The fluorescence signal intensities were read, log-transformed and corrected for the signal from the polyclonal secondary antibody binding directly to the peptide probes. Subsequently, the signal intensities were mean-centered and scaled to unit variance, which resulted in a normalized vector $\vec{s}$ for each IgM and IgG serum sample and for each of the 13 monoclonal antibodies. We use the terms signal intensity or antibody binding profile interchangeably to denote $\vec{s}$. Each signal intensity vector $\vec{s}$ has as many components as there are peptides in the standard random peptide libary. For brevity, our analysis focuses on the IgM data. The IgG data can be found in the Supporting Information (Additional file 2, Figure S2, Additional file 3, Figure S3, and Additional file 4, Figure S4). More details on the experimental setup and normalization procedures can be found in *Methods*.

### A regression model based exclusively on peptide amino acid composition predicts antibody binding profiles

We built a linear statistical model to relate the amino acid composition of our peptide library to measured signal intensities

(1) $$\vec{s}=X\vec{w}+\vec{\varepsilon }\text{,}$$

where $\vec{s}\left(255\times 1\right)$ is the signal intensity vector and **X **the amino acid composition matrix (AACM) of the peptide library. The **X **matrix is formed by counting the occurrences of each of the 20 amino acids in each peptide which results in a matrix with 20 columns and 255 rows. Importantly, **X **does not contain information about the position of an amino acid in a given peptide sequence.

The AAWS vector $\vec{w}\left(20\times 1\right)$ indicates the contribution of every amino acid to the measured signal intensity. Furthermore, the residual of the regression model, $\vec{\varepsilon }$, captures the part of $\vec{s}$ which cannot be explained by **X **alone. AAWS and residuals were estimated by partial least squares regression (PLS) (see *Methods *for details on the data analysis).

**Figure 1:**
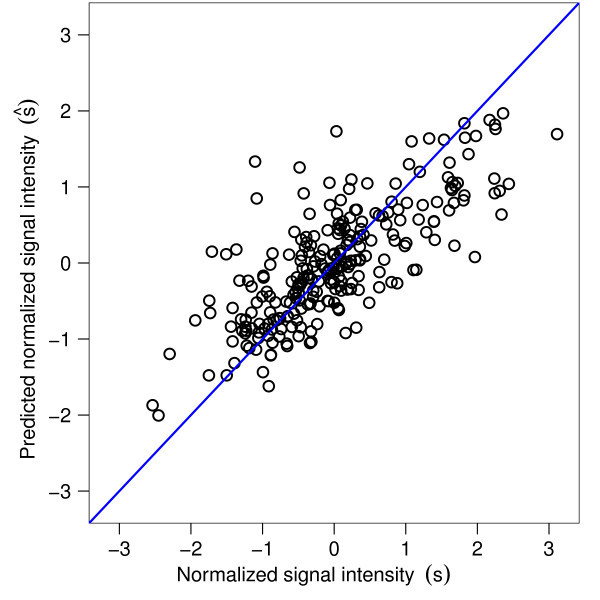
**Predicted signal intensity ($\hat{\vec{s}}$) against measured signal $\vec{s}$ of a healthy BALB/c mouse serum sample**. The prediction depends exclusively on the amino acid composition of the peptide sequences and is based on the regression model (Equation 1). Predictive performance: *Q*^2 ^= 0.5. Signal intensities were measured with the standard peptide library of 255 14-mers.

Once the vector $\vec{w}$ has been estimated, we use the regression model to predict measured signal intensities given the peptides' amino acid composition. Figure 1 illustrates that the predicted signal intensities $\hat{\vec{s}}=X\vec{w}$ are in good agreement with signal intensities $\vec{s}$ measured for the serum of one healthy BALB/c mouse. In order to evaluate the performance of the regression model, we focus on the predictive performance, *Q*^2^, which was determined by 10-fold cross-validation (*Methods*). The predictive performance equals 1 for perfect predictions and is close to zero for poor predictions.

**Figure 2:**
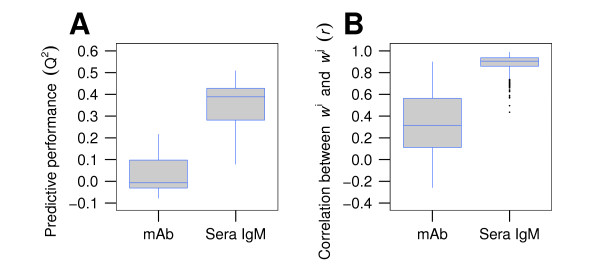
**Predictive performance and pairwise correlation of AAWS are higher for serum IgM than for monoclonal antibodies**. (A) Predictive performance values were calculated for monoclonal (mAb) and serum IgM antibody (Sera IgM) binding profiles. (B) Shown is the pairwise correlation (*r*) of the corresponding AAWS ${\vec{w}}^{j}$. In both (A) and (B), mAb signifies antibody binding profiles from 13 monoclonal antibodies and Sera IgM binding profiles from 58 BALB/c mouse serum samples. Differences between monoclonal and serum IgM antibodies in predictive performance (*Q*^2^) and pairwise correlation (*r*) of AAWS are significant (*p *< 0.001). Antibody binding profiles were measured with the standard peptide library of 255 14-mers. Corresponding AAWS (${\vec{w}}^{j}$) were determined using Equation 1.

All 58 BALB/c serum samples resulted in a median predictive performance of 0.39 (Figure 2).

### A minimal model of antibody-peptide binding

We hypothesize that the high predictive performance of our regression model is due to properties of an antibody ensemble. We test this hypothesis with the help of a model that simulates binding between peptides and antibodies. In this model, the binding affinity of simulated monoclonal antibodies depends non-linearly on amino acid positions in the peptide sequences (Equations 2 and 4). The model we propose is similar to bit string models [23-26] in that it uses vectors as simple representations of peptides and antibodies. The peptide string is represented by unique real numbers taken from a vector of assigned AAWS, denoted $\vec{h}$, the twenty components of which were drawn from a uniform distribution on the closed interval 0[1]. A peptide $\overset{\to }{p^{i}}$ of *l *amino acids is thus represented by a vector of *l *numbers drawn from $\vec{h}$.

An antibody binding site is represented by a vector $\overset{\to }{a^{k}}$ of length *l*. The binding strength of each position is given by a number between -1 and 1 that is drawn randomly from a uniform distribution and is scaled such that $\overset{\to }{{\left(a^{k}\right)}^{\text{T}}}\overset{\to }{a^{k}}=1$. The binding association between peptide $\overset{\to }{p^{i}}$ and antibody $\overset{\to }{a^{k}}$ is computed as the dot product of the two vectors, $y_{i,k}={\overset{\to }{{(a}^{k})}}^{\text{T}}\overset{\to }{p^{i}}$. Thus, the binding association *y*_*i, k *_depends explicitly on an amino acid's position in a given peptide sequence.

An expression for the simulated signal intensity, based on the law of mass action, can be obtained from classical Langmuir adsorption theory [27]:

(2) $$S_{i}=\frac{\sum_{k=1}^{n_{\text{Ab}}}{\left[\text{Ab}\right]}_{k}K_{i,k}}{1+\sum_{k=1}^{n_{\text{Ab}}}{\left[\text{Ab}\right]}_{k}K_{i,k}}$$

where [Ab]*_k _*is the concentration of antibody *k *with $\sum_{k=1}^{n_{\text{Ab}}}{\left[\text{Ab}\right]}_{k}=1$. The thermodynamic equilibrium association constant for antibody *k *binding peptide *i *is defined as $K_{i,k}=\text{exp}\left(-\frac{{\text{Δ}}_{r}G^{\text{o}}}{RT}\right)$ with ${\text{Δ}}_{r}G=\text{exp}\left(\frac{\beta_{0}+\beta_{1}y_{i,k}}{RT}\right)$. Logarithmizing the results of Equation 2, and centering them to zero and unit variance, we obtained a vector of normalized simulated signal intensities ${\vec{s}}_{\text{sim}}$. A more detailed description of the mathematical model can be found in *Methods*.

### Simulations show that the prediction of antibody binding profiles based exclusively on peptide amino acid composition improves with increasing antibody diversity

**Figure 3:**
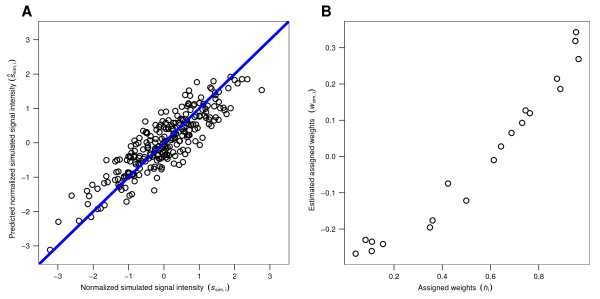
**Simulated signal intensities and assigned amino acid-associated weights are recovered by an amino acid composition-based regression model**. (A) Simulated signal intensities (${\vec{s}}_{\text{sim}}$) obtained by amino acid position-dependent simulation of the binding of 150 antibodies to an array of 255 14-mer peptides were predicted using the regression model given by Equation 1. This regression model takes into account only the amino acid composition of the simulated peptide sequences. Predictive performance: *Q*^2 ^= 0.40. Simulated signal intensities were computed using Equation 2. (B) Equation 1 was used to estimate (${\vec{w}}_{\text{sim}}$), which are shown against the assigned AAWS ($\vec{h}$) used to generate the simulated signal intensities ${\vec{s}}_{\text{sim}}$. Coefficient of correlation: *r *= 0.92.

We first simulated signal intensities for *n*_Ab _= 150 binding to a simulated peptide library of 255 14-mers. The peptide library used in the simulation determines the amino acid composition matrix **X**_sim_. We estimated simulated intensities ${\vec{s}}_{\text{sim}}$ (Figure 3A) and respective weights ${\vec{w}}_{\text{sim}}$ (Figure 3B) using the linear regression model ${\hat{\vec{s}}}_{\text{sim}}=X_{\text{sim}}w_{\text{sim}}^{\to }$. Prediction of simulated signal intensities yielded a predictive performance (*Q*^2^) of 0.40, and the correlation between $\vec{h}$ and ${\vec{w}}_{\text{sim}}$ was found to be *r *= 0.92 (Figure 3B), which indicates a very good recovery of $\vec{h}$. Recall that signal intensities were simulated in an amino acid position-dependent manner, while the composition-based regression model (Equation 1) relies on the amino acid position-independent matrix **X**_sim_.

**Figure 4:**
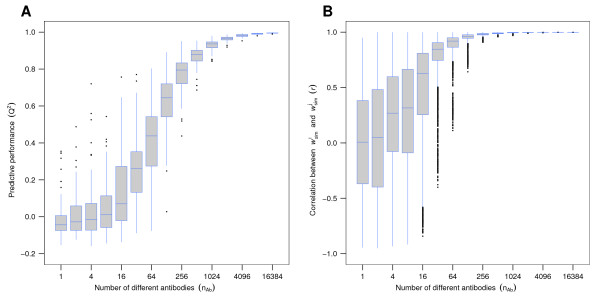
**Simulations show that predictive performance of antibody binding profiles improves with increasing antibody diversity**. Antibody binding profiles (${\vec{s}}_{\text{sim}}^{i}$) were simulated for antibody mixtures of 1 to 16384 different antibodies. (A) Predictive performance increases with increasing number of antibody variants (*n*_Ab_), (B) as does the correlation (*r*) between all pairs of predicted AAWS ${\vec{w}}_{\text{sim}}^{i}$. In both (A) and (B), a simulated random peptide library (**X**_sim_) of 255 14-mers and assigned AAWS ($\vec{h}$) were generated once and were kept constant across all simulation runs. Notably, varying **X**_sim _for every simulation run did not change either of the boxplot distributions. For every mixture of *n*_Ab_-different antibodies, 100 simulations with newly generated random antibody mixtures were run. Antibody binding profiles were computed using Equation 2. Corresponding AAWS (${\vec{w}}_{\text{sim}}^{i}$) were determined using Equation 1.

Further, our simulation framework enabled us to show *in silico *that the predictive performance increases with growing antibody diversity (Figure 4A). The same is true for the pairwise correlation of computed AAWS $\left({\vec{w}}_{\text{sim}}^{i}\right)$, which nears perfection (*r *= 1) with increasing antibody diversity (Figure 4B), as does the correlation of AAWS with $\vec{h}$ (Additional file 5, Figure S5). Therefore, when using a position-independent linear statistical model for the prediction of antibody-peptide binding, high antibody diversity is a prerequisite for good predictive performance.

### Predictive performance differs for monoclonal and serum-antibody binding profiles

In order to test our *in silico*-based prediction that predictive performance depends heavily on antibody diversity when only taking into account the peptide library's amino acid composition, we compared the predictive performance of the 58 BALB/c mouse serum samples (antibody diversity *n*_Ab _>> 1) with that of the 13 human monoclonal IgG antibodies (antibody diversity *n*_Ab _= 1). We found both a significantly higher predictive performance (Figure 2A, p < 0.001) and significantly higher pairwise correlations between AAWS for serum antibodies (Figure 2B, p < 0.001) than for monoclonal antibodies, which confirms the predictions of our mathematical model (Figure 4).

### Predictive performance decreases in the course of an HB-infection

**Figure 5:**
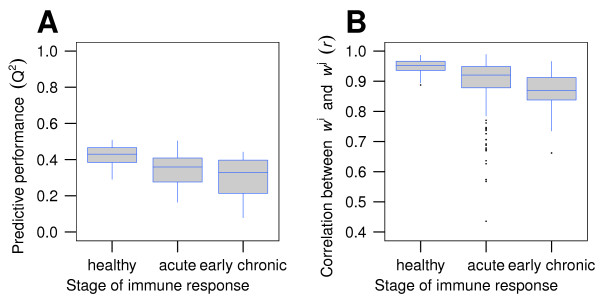
**Predictive performance and pairwise correlation of AAWS decrease for serum IgM antibodies in the course of the HB-infection**. (A) Predictive performance values (*Q*^2^) were computed from serum IgM antibody binding profiles across three stages of immune response: *healthy, acute, early chronic*. (B) Shown is the pairwise correlation (*r*) of the corresponding AAWS ${\vec{w}}^{j}$. Numbers of BALB/c mouse serum samples: 15 samples from *healthy *mice; after infection with HB: 15 samples at 10 dpi and 13 samples at 14 dpi (*acute phase*), and 15 samples at 18 dpi (*early chronic*) totaling 58 BALB/c mouse serum samples. Differences in predictive performance (*Q*^2^) between *healthy *and both *acute phase *and *early chronic phase *mice are significant (*p *< 0.01), as are differences in pairwise correlation (*r*) between all three stages of immune response (*p *< 0.001). Antibody binding profiles were measured with the standard peptide library of 255 14-mers. Corresponding AAWS (${\vec{w}}^{j}$) were computed using Equation 1.

In order to quantify the influence of immune response stage during HB-infection on predictive performance, we divided the mouse serum samples into three groups: *healthy, acute phase *(10 and 14 dpi), and *early chronic phase *(18 dpi) [28]. We found that predictive performance (Figure 5A) and pairwise correlation of AAWS decrease significantly in the course of the immune response (Figure 5B).

**Figure 6:**
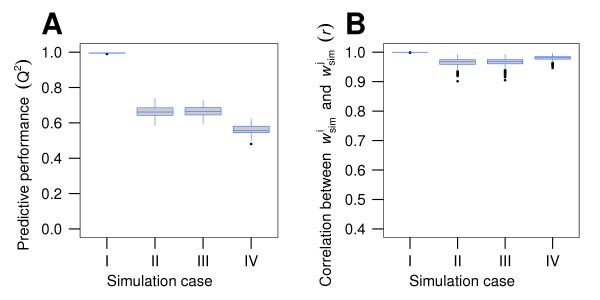
**Predictive performance and pairwise correlation of simulated AAWS decrease both with introduction of multiplicative Gaussian noise and antibody dominance**. (A) Predictive performance (*Q*^2^) and (B) pairwise correlation of AAWS (*r*) for different simulated cases. (I) For a given peptide library and given assigned AAWS ($\vec{h}$) we simulated 100 realizations of binding profiles for a mixture of 16000 different antibodies. (II) Same as in I, but Gaussian multiplicative noise was introduced into the simulated signal intensities. (III) Same as in II, but the concentration of a single antibody (dominant antibody) was increased 10-fold. (IV) Same as in II, but concentration of one (dominant) antibody was increased 1000-fold. For both (A) and (B) a simulated peptide library (**X**_sim_) and assigned AAWS ($\vec{h}$) were generated once and kept constant across the entire simulation. Simulated antibody binding profiles (${\vec{s}}_{\text{sim}}^{i}$) were computed using Equation 2. Corresponding AAWS (${\vec{w}}_{\text{sim}}^{j}$) were computed using Equation 1. In each of the 100 runs, a newly generated random antibody mixture of *n*_Ab _= 15999 different antibodies was simulated to which the dominant antibody was added. This antibody was randomly generated once at the beginning of the simulation and was kept constant across all four simulation cases. Gaussian noise term: N(*μ *= 0, *σ *= 0.01).

In order to compare the experimental results with the mathematical model, we simulated signal intensities for 100 random mixtures of 16000 different antibodies (Figure 6A and 6B, case I) and found that, when multiplicative Gaussian noise is introduced into the simulated signal intensities, both predictive performance and pairwise correlation of AAWS decrease (Figure 6A and 6B, case II). By increasing the concentration of one monoclonal antibody (the dominant antibody) to a sufficiently high level (Figure 6A and 6B, cases III and IV), predictive performance is decreased.

### Stages of murine immune response differ in their amino acid-associated weights

**Figure 7:**
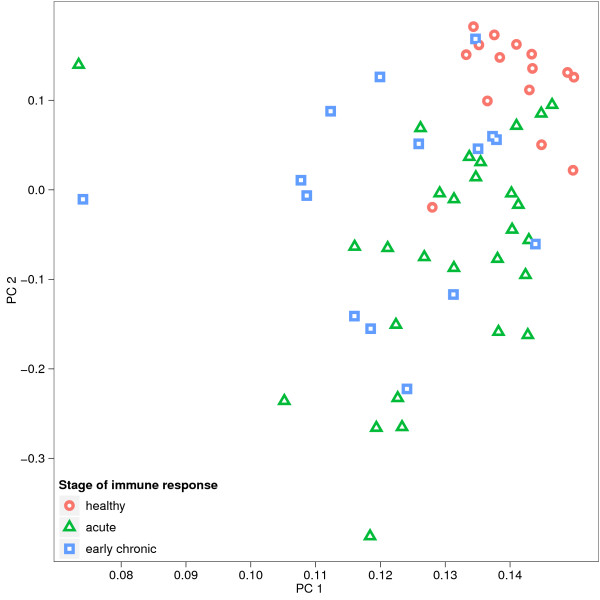
**Stages of immune response differ in their AAWS**. 58 BALB/c mouse serum samples were put into three groups, each corresponding to one stage of immune response: *healthy, acute *and *early chronic*. Serum IgM antibody binding profiles were determined using the standard peptide library consisting of 255 14-mers. Corresponding AAWS (${\vec{w}}^{j}$) were determined with Equation 1. AAWS were then projected by principal component analysis onto a 2-dimensional subspace spanned by the first two principal components (PC1, PC2). Together, the first two principal components yield a strong separation of *healthy *and diseased mice. Also *acute *and *early chronic *samples separate, although less clearly. The proportion of variance explained by PC1 and PC2 is 90% and 3.4%, respectively. Number of BALB/c mouse serum samples: 15 from *healthy *mice; after infection with HB: 15 samples at 10 dpi and 13 samples at 14 dpi (*acute phase*), and 15 samples at 18 dpi (*early chronic*).

In order to test whether the AAWS determined for all 58 BALB/c mouse serum samples were systematically different from one another, we applied principal component analysis to them. Together, the first two principal components yield a strong separation of *healthy *and diseased mice. Also, *acute *and *early chronic *samples separate (Figure 7). Thus, during an immune response against HB, AAWS change in a systematic way.

### Average amino acid-associated weights of healthy mice correlate with amino acid physico-chemical properties but not with widely used amino acid scales for epitope prediction

**Figure 8:**
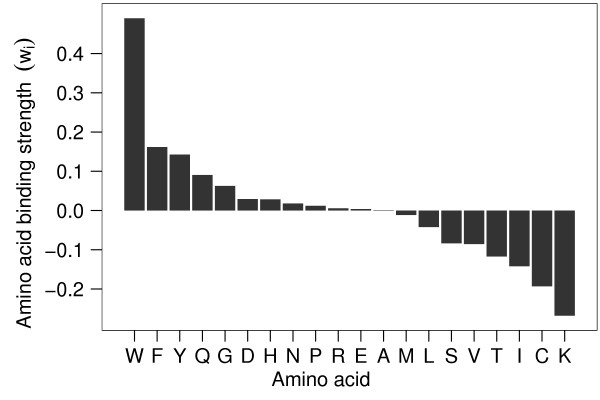
**Average recognition of peptides by serum samples of healthy BALB/c mice as given by their AAWS**. AAWS of the 15 healthy BALB/c mice (Figure 5A, B; left boxplot) were determined with Equation 1 and averaged. The difference between two weights indicates the contribution to the difference in normalized signal intensity corresponding to an amino acid substitution.

Because of both the good predictive performance and the high pairwise correlation of AAWS of healthy BALB/c mice, we considered their average AAWS as representative of healthy BALB/c mice (Figure 8). The differences between weights in Figure 8 indicate the difference in contribution to normalized signal intensity corresponding to an amino acid substitution. Tryptophan, phenylalanine and tyrosine, all of which have aromatic residues, contribute most to the signal intensity.

AAWS represent a priority scale for peptide-antibody binding assigning to every amino acid the importance of contribution to the measured (or simulated) signal intensity. In addition, analogously to QSAR modeling, AAWS can a posteriori be conceived of as a vector representing correlates of the respective amino acids' physico-chemical properties. We therefore correlated the average AAWS (Figure 8) with the z-scale developed by Sandberg and colleagues [29]. The z-scale aggregates in matrix form 26 physico-chemical amino acid properties for every one of the 20 examined amino acids (Additional file 6, Figure S6). The average AAWS yield an absolute correlation coefficient higher than 0.3 with the following physico-chemical properties: *side chain van der Waals volume, alpha-polarizability, absolute electronegativity, number of hydrogen bond donors, total accessible molecular surface area, and indicator of negative charge in side chain*.

In order to compare the average AAWS with other published amino acid-scales for epitope prediction, we correlated them with propensity scales published by Parker and colleagues [17] (hydrophilicity), Kolaskar and Tongaonkar [30] (antigenicity), Chou and Fasman [16] (secondary structure) and by Emini and colleagues [18] (accessibility) and found the resemblance with them to be poor (absolute values of correlation coefficients smaller than 0.22). Notably, the compared propensity scales also do not highly correlate (range of correlation coefficients: -0.61 to 0.67).

## Discussion

### Amino acid-associated weights are a compact, information-preserving representation of serum-antibody binding profiles

A minimal linear regression model defines AAWS as predictors that are based solely on the amino acid composition of a given peptide. For serum antibodies of BALB/c mice, AAWS account for up to 50% of variation in antibody binding profiles, whereas monoclonal antibodies generally show poor predictive performance values. The regression model performs best for healthy mice (median *Q*^2 ^= 0.43, Figure 5). Furthermore, we find AAWS to be comparable across healthy BALB/c mouse serum samples (Figure 5B). During the immune response against HB, however, predictive performance decreases steadily. Accordingly, pairwise correlations of AAWS are highest for healthy mice and decrease during the immune response (Figure 5). Therefore, we hypothesize that the average AAWS for healthy mice, shown in Figure 8, are a signature of health. AAWS of infected mice, in turn, are systematically different from AAWS of healthy mice and can be separated by principal component analysis.

### Simulated unbiased antibody mixtures show ensemble properties

In order to interpret the reported experimental results, we built a mathematical model based on the law of mass action. We defined a property vector $\vec{h}$ that characterizes each peptide's amino acid binding strength. In this model, the binding signals for a given simulated monoclonal antibody depend on the amino acid's position in a given peptide.

For a single simulated antibody, AAWS calculated by the amino acid composition-based linear regression model generally yield neither good predictive performance nor a high correlation with assigned AAWS $\vec{h}$. However, highly diverse antibody mixtures with random--in the sense of an independent identically distributed--repertoire, and no dominant antibodies, show both a close to perfect predictive performance and recovery of assigned AAWS $\vec{h}$ (Figure 4 and Additional file 5, Figure S5). Our mathematical model thus predicts that high predictive performance and high correlation of estimated AAWS and $\vec{h}$ are ensemble properties of such antibody mixtures: the average affinity of these mixtures does not depend on the epitope's amino acid position anymore. In contrast to that, the monoclonal antibody-epitope affinities do. We call random and highly diverse antibody mixtures that are not biased by dominant antibodies "unbiased". In fact, introducing, in simulations, a dominant antibody by increasing the concentration of a single antibody decreases predictive performance (Figure 6A). In addition, we showed that noise reduces predictive performance (Figure 6A).

### Serum samples of healthy BALB/c mice show signs of unbiased antibody mixtures

As shown in our mathematical model, unbiased antibody mixtures are characterized by high predictive performance values. In view of the relatively high predictive performance of antibody binding profiles of serum samples from healthy BALB/c mice, we postulate that these sera exhibit properties of unbiased antibody mixtures.

The first prerequisite for an unbiased mixture is high diversity. This requirement seems to be met. The potential antibody diversity is very high [31], and the functional diversity is estimated to be of the order of 10^4 ^[32]. However, fulfillment of the second requirement, the independent identical distribution of antibody binding sites, is harder to claim. On the one hand, the antibody repertoire is composed of preexisting gene segments and shaped by clonal selection, but on the other hand, V(D)J recombination and--in later stages of an immune response--somatic hypermutation arrange and mutate these segments in a largely random fashion [1]. Our results suggest that randomness in fact prevails. This is consistent with the hypothesis that antibody repertoires can potentially recognize the entire antigenic universe [33,34].

The predictive performance of healthy BALB/c mice is not perfect but amounts to a median of 0.43. This can be due to both noise and the fact that serum violates the assumptions of randomness to a certain degree. Noise may be caused by varying peptide spot quality on microarrays and by the experimental procedure itself. It is known that during a primary acute immune response, antibodies of a certain specificity for the antigen are produced in high abundance [35,36]. Therefore, it can be expected that sera of infected mice deviate from the properties of an unbiased mixture and would have reduced predictive performance values. Indeed, this is corroborated by experimental results (Figures 5 and 7).

### Unbiased mixtures represent a special case for which the use of propensity scales for epitope prediction is justified

The prediction of linear B-cell epitopes was first done by using propensity scales [19,37,38]. These scales assign a propensity value to every amino acid based on a priori studies of their physico-chemical properties. We found that our average AAWS, a posteriori termed signature of health (Figure 8), are not significantly correlated to widely used propensity scales.

Blythe and Flower tested 484 amino acid propensity scales on a set of 50 epitope-mapped proteins. They found that even the best set of scales perform only marginally better than random [39]. We show that unbiased mixtures represent a special case for which the converse holds true: antibody binding profiles of unbiased mixtures can be predicted based on AAWS. We show that the use of amino acid scales becomes increasingly less justified with increasing dominance of antibodies in a serum. In fact, each of Blythe and Flower's experiments used polyclonal antibodies raised against the whole protein [39]. We conjecture that the used polyclonal antibody mixtures were biased, that is, they contained dominant antibodies. In this regard, our study provides a possible explanation to Blythe and Flower's survey. More generally, our work suggests that results obtained with polyclonal antibody mixtures tend to be skewed by the inherent ensemble properties, which obscure the affinities of epitope-specific antibodies.

### Technological features may bias amino acid-associated weights

We have shown that antibody mixtures exhibit ensemble properties. Resulting AAWS were shown to be consistent across healthy mice and qualitatively different from AAWS of infected mice (Figure 7). We have also provided a possible explanation for the difference between AAWS of healthy and infected mice: dominant antibodies in the course of the immune response.

However, the actual signature of health values shown in Figure 8 should be interpreted with caution. In addition to being indicative of both amino acid antibody binding preferences and physico-chemical properties (Figure 8 and Additional file 6, Figure S6), signal intensity may also be influenced by two other factors: (i) the accessibility of peptides and (ii) a possible interaction of aromatic amino acids and aromatic labeling dyes.

Accessibility may bias the resulting signal intensities systematically. For example, we find that cysteine contributes negatively to the signal intensity. This could partly be due to its ability to form disulfide bonds, which causes increased aggregation of cystein-containing peptides, and diminishes their surface exposure. This would lead to reduced antibody-peptide binding and accordingly to reduced signal intensity. Furthermore, it cannot be ruled out that aromatic amino acids interact via *π*-stacking with the aromatic labeling dyes Alexa Fluor 546 and 647 which are coupled to the secondary antibodies. Indeed, it has recently been found that TAMRA, another aromatic dye, cross-reacts with individual amino acids in a peptide sequence [40]. In order to minimize this effect, we performed secondary antibody correction on the log-transformed signal intensities.

## Conclusions

We show that due to ensemble properties of unbiased mixtures, the position of amino acids in a linear epitope is no longer determinative for binding prediction. We found that prediction of peptide-binding as well as consistence of AAWS was best in sera of healthy BALB/c mice. Therefore, we defined a signature of health characterizing the binding behavior of serum of healthy individuals. This finding has far-reaching significance for the field of serological diagnostics.

Furthermore, our findings have also deep implications for the field of B cell epitope mapping as we have discovered an important special case which enables amino acid scale prediction of peptide binding. We show that amino acid scale prediction of peptide binding is justified only for unbiased mixtures. For other cases, alternative methods have to be sought. We thus showed that a knowledge of the composition of the used polyclonal mixture is essential for both the choice of the prediction method as well as the interpretation of results.

In the future, it will be of great interest to investigate the effects of a more detailed representation of binding in the mathematical model, and to study the effect of non-uniform antibody concentration distributions on predictive performance. Indeed, it has recently been shown for healthy zebrafish that the B cell clone repertoire follows a power-law distribution [41]. Thanks to our minimal assumptions approach, the conclusions of our model are independent of species, genetical background and individual exposure history. Future studies have to verify these predictions.

## Methods

### Ethics Statement

Animals were housed and handled following national guidelines and as approved by our animal ethics committee.

### Mice

BALB/c mice were bred and maintained under specific pathogen-free (SPF) conditions by the Department of Molecular Parasitology, Humboldt University Berlin, Berlin, Germany. Infection of mice with HB was carried out by oral gavage with 200 L3 stage larvae in distilled water.

### Sera

Mice were narcotized and bled either by cardiac or retro-orbital puncture at the age of 8 weeks. Blood samples were collected from healthy SPF-BALB/c mice (*n *= 15), which were then infected with HB. Blood was collected at three time points post infection (dpi): at 10 dpi (*n *= 15), 14 dpi (*n *= 13) and 18 dpi (*n *= 15). The blood was allowed to clot at room temperature and centrifuged. The supernatant was stored at -20°C.

### Monoclonal antibodies

The 13 human monoclonal antibodies were kindly provided by the group of Hedda Wardemann (Max Planck Institute for Infection Biology, Berlin, Germany). Ten different Ig gene sequences of IgG^+ ^memory B cells from 2 healthy human donors, PN and VB, (PN115, PN138, PN16, PN89, VB1, VB142, VB161, VB176, VB18, VB4) [42] and three further ones from 3 other human donors ED38 [43], eiJB40 and mGO53 [44] were expressed as detailed in [45].

### Random peptide library

The peptide library consists of 255 different 14-mer peptides. Their sequence was designed with a random generator. Repetitions of three or more consecutive amino acids were not allowed.

### Peptide synthesis and microarray design

The peptide library was displayed in five identical sub-arrays on each slide purchased from JPT Peptide Technologies GmbH, Berlin, Germany. Furthermore, TAMRA-derived peptides, as internal fluorescence control, and mouse-IgM, mouse-IgG, human-IgM and human-IgG as secondary antibody controls, were included on each sub-array. Peptide microarrays were stored at 4°C.

### Antibody binding assays

The microarrays were briefly immersed in 100% v/v ethanol, washed three times with T-PBS (phosphate buffered saline containing 0.05% w/v Tween20), three times with deionized water and dried by centrifugation. Since the microarray surfaces had been pre-treated to minimize unspecific binding of the target antibodies, no blocking step was required prior to incubation. All incubations were performed using a five-well adhesive incubation chamber (Multiwell GeneFrameTM, ABgene Germany, Hamburg, Germany) with a total assay volume of 45*μ*L per well. Serum was diluted 1:10 in T-PBS and monoclonal antibodies were applied in a concentration of 10*μ*g/mL. We showed in a technological case study that approximately 10*μ*g/ml of antibody are best for reliable signal intensity measurements [14]. The concentration of IgM in in the serum of healthy SPF BALB/c mice was found to be around 0.50 mg/ml [46], which yields 50*μ*g/ml for a 1:10 dilution. The diluted sera are thus within the optimal binding range. After incubation for four hours at room temperature, the microarrays were washed three times with T-PBS and three times with deionized water. Serum-antibody binding was detected with polyclonal goat anti-mouse IgM-Alexa Fluor 546 and polyclonal goat anti-mouse IgG-Alexa Fluor 647 (Invitrogen Ltd, Paisley, UK), simultaneously.

Monoclonal antibody binding was detected with polyclonal goat anti-human IgG Alexa Fluor 647 (Invitrogen Ltd, Paisley, UK). Secondary antibodies were diluted in T-PBS (20*μ*g/mL, 300*μ*L) and incubated for one hour at room temperature. The microarrays were washed three times with T-PBS, three times with deionized water, rinsed with running deionized water and dried by centrifugation. Water, ethanol and PBS were filtered.

### Signal detection

Fluorescence signals were measured on a GenePix microarray scanner (Molecular Devices GmbH, Ismaning, Germany) with a 532 nm laser using green (~ 550-600 nm) emission filters and with 635 nm laser using red (~ 650-690 nm) emission filters. An image file was generated at a resolution of 10*μ*m using the scanner-associated GenePix^® ^Pro software. Signal intensities were quantified with Genespotter™ software (MicroDiscovery GmbH, Berlin, Germany). Genespotter provides a fully automated grid-finding function, resulting in a reproducible read-out procedure. Signal intensities for each spot were calculated from a circular region around the center of the spot. Spots were examined for auto-fluorescence, but no relevant correlation between peptide composition and the fluorescence of clean microarrays was observed. Measured raw signal intensities were logtransformed (log(I)). Subsequently, the signal arising from the polyclonal secondary antibody was removed according to the linear model:

(3) $$\text{log}\left(I\right)=\beta_{0}+\beta_{1}\text{log}\left(I_{SecondaryAntibody}\right)+\varepsilon \text{.}$$

By PLS-based computation of the intercepts, *β*_0 _and *β*_1_, we replaced log(*I*) with the resulting PLS-computed, mean-centered and scaled-to-unit variance residuals *ε *for further analysis. The results reported in the main text of this paper are based exclusively on the calculated normalized residuals.

### Statistics

The two-sided, non-paired Wilcoxon rank sum test was used to compute all p-values. P-values were regarded as significant when *p *< 0.05. Association between variables was assessed by Pearson correlation (*r*) unless otherwise stated.

### Generation of simulated signal intensities with a mathematical model

Peptides and antibody binding sites were modeled as strings. Binding strengths between antibodies and the various amino acid residues of a peptide, referred to as assigned AAWS $\vec{h}$, were sampled from the uniform distribution on the closed interval 0[1]. A binding site on an antibody $\overset{\to }{a^{k}}$ was simulated in a similar fashion with a random number from the closed interval [-1, 1] for every sequential position and scaled such that ${\overset{\to }{\left(a^{k}\right)}}^{\text{T}}\overset{\to }{a^{k}}=1$. The binding association between peptide $\overset{\to }{p^{i}}$ and antibody $\overset{\to }{a^{k}}$ was calculated by $y_{i,k}={\overset{\to }{{(a}^{k})}}^{\text{T}}\overset{\to }{p^{i}}$.

Based on the interpretation of the binding association as being negatively linearly proportional to the standard Gibbs free energy change of reaction, Δ*_r_G*^o^, the binding affinity *K*_*i, k*_, that is, the thermodynamic equilibrium association constant for antibody *k *binding peptide *i*, is defined as shown in Equation 4.

(4) $$K_{i,k}=\text{exp}\left(-\frac{\Delta_{r}G^{\text{o}}}{RT}\right)=\text{exp}\left(\frac{\beta_{0}+\beta_{1}y_{i,k}}{RT}\right)$$

Similar to a bit string model approach in [47], our approach to calculating *K*_*i, k *_assumes additivity in free energy of binding, an assumption that is supported by experimental results [48,49]. The signal intensity that we measure on the array is assumed to be proportional to the ratio of bound-to-total surface of the peptide spot, *S_i_*. An expression for this quantity, based on the law of mass action, can be obtained from classical Langmuir adsorption theory [27] resulting in Equation 2 with *R *= 8.314472, *T *= 273.15 + 25, *β*_0 _= 0 and *β*_1 _= *RT*.

At last, signal intensities were log-transformed, mean-centered, and scaled to unit variance. If Gaussian noise (N(*μ *= 0, *σ *= 0.01)) was introduced into simulated signal intensities, the noise term was introduced before logarithmic transformation of the data. We showed that, for monoclonal antibodies, visibly fluorescent spots have at least a *K*-value of 10^7^M^-1 ^[14].

### Partial least squares regression

All calculations involving PLS were carried out with the pls package [50] for the R statistical programming environment [51].

### Model diagnostics

The predictive performance is defined as:

(5) $$Q^{2}=1-\frac{\sum {\left({ŝ}_{\text{Leftout}}-s_{\text{Leftout}}\right)}^{2}}{\sum^{\text{​}}s_{\text{Leftout}}^{2}}.$$

The vector ${\vec{s}}_{\text{Leftout}}$ is the left-out test data set, the signal intensity of which is predicted (${\hat{\vec{s}}}_{\text{Leftout}}$) from the remaining training data set. The left-out test data represented randomly chosen 10% of the total data set.

### Principal component analysis

Principal component analysis was performed using the pcaMethods R-package [52].

## List of Abbreviations

AACM: Amino acid composition matrix; AAWS: Amino acid-associated weights; FABR: Functional antibody repertoire; HB: *Heligmosomoides bakeri*; PLS: Partial least squares regression.

## Authors' contributions

MOG conceived of the project. HR, VG and MOG designed the research. HR, JS, VG and MOG analyzed data. HR, VG and MOG wrote the manuscript. NB and JL performed peptide array experiments. SR and SH provided murine sera. All authors discussed results. All authors have read and approved the final manuscript.

## Supplementary Material

Additional File 1 **Supporting Figure S1: Experimental setup: infection of BALB/c mice with *Heligmosomoides bakeri *and collection of blood samples at three different stages of immune response**. Serum samples from 15 BALB/c mice raised under specific pathogen-free conditions were collected. These mice were infected with the intestinal nematode *Heligmosomoides bakeri *formerly known as *Heligmosomoides polygyrus *[53]. Further serum samples were collected at 10 dpi (days post infection; 15 samples), at 14 dpi (13 samples), and at 18 dpi (15 samples) totaling 58 serum samples. The serum was isolated and subsequently incubated with random peptide libraries. We categorized the serum samples into *healthy *(0 dpi; 15 samples), *acute phase *(10 and 14 dpi; 15 and 13 samples respectively) and *early chronic phase *(18 dpi; 15 samples), thus delineating the three major stages of immune response of a mouse, before and after primary infection with HB. Practical experimental difficulties reduced the intended number of usable 14 dpi samples from 15 to 13. Click here for file

Additional File 2 **Supporting Figure S2: Removing the signal of the secondary antibody accentuates differences between binding profiles of monoclonal and serum antibodies**. (A) The predictive performance values (*Q*^2^) were calculated for monoclonal (mAb) as well as serum IgM (sIgM) and IgG (sIgG) antibody binding profiles before (blue) and after (red) correction of the measured log-transformed signal intensities by removal of the polyclonal secondary antibody-correlated signals using PLS. (B) Shown is the pairwise correlation (*r*) of the corresponding AAWS ${\vec{w}}^{j}$. For the two statistical measures, signal correction entails a significant decrease in the mAb median, whereas sIgM and sIgG medians remain largely unchanged. Both before and after secondary antibody correction of antibody binding profiles, sIgM profiles have higher predictive performance (*Q*^2^) and a higher median pairwise correlation (*r*) of AAWS than sIgG profiles. In (A) and (B), mAb signifies antibody binding profiles from 13 monoclonal antibodies and sIgM/sIgG serum IgM and serum IgG binding profiles from 58 BALB/c mice sera, respectively. Antibody binding profiles were measured with the standard peptide library of 255 14-mers. Corresponding AAWS (${\vec{w}}^{j}$) were determined with Equation 1. Click here for file

Additional File 3 **Supporting Figure S3: Predictive performance and pairwise correlation of amino acid-associated weights are higher for serum IgG than for monoclonal antibodies**. (A) Predictive performance values (*Q*^2^) were calculated for monoclonal (mAb) and serum IgG antibody (Sera IgG) binding profiles. (B) Shown is the pairwise correlation (*r*) of the corresponding AAWS ${\vec{w}}^{j}$. In both (A) and (B) mAb signifies antibody binding profiles from 13 monoclonal antibodies and Sera IgG binding profiles from 58 BALB/c mice sera. Differences in predictive performance (*Q*^2^) and pairwise correlation (*r*) of AAWS between monoclonal and serum IgG antibodies are significant (*p *< 0.001). Antibody binding profiles were measured with the standard peptide library of 255 14-mers. Corresponding AAWS (${\vec{w}}^{j}$) were determined with Equation 1. Click here for file

Additional File 4 **Supporting Figure S4: Predictive performance and pairwise correlation of amino acid-associated weights decrease for serum IgG antibodies during the course of the immune response**. (A) Predictive performance values (*Q*^2^) were computed from serum IgG antibody binding profiles across three stages of immune response: *healthy, acute, early chronic*. (B) Shown is the pairwise correlation (*r*) of the corresponding AAWS ${\vec{w}}_{\text{sim}}^{j}$. Number of BALB/c mice serum samples: 15 from *healthy *mice, after infection with HB: 15 samples taken at 10 dpi and 13 samples taken at 14 dpi (*acute phase*) and 15 samples taken at 18 dpi (*early chronic*) totaling 58 BALB/c serum samples. Differences in predictive performance (*Q*^2^) between both *healthy *and *early chronic phase *mice and *healthy *and *acute phase mice *are significant (*p *< 0.05) as are differences in pairwise correlation (*r*) between all three stages of immune response (*p *< 0.001). Antibody binding profiles were measured with the standard peptide library of 255 14-mers. Corresponding AAWS (${\vec{w}}^{j}$) were computed using Equation 1. Click here for file

Additional File 5 **Supporting Figure S5: Simulations show that recovery of assigned amino acid-associated weights is positively correlated to antibody diversity**. This Figure is complementary to Figure 3. Antibody binding profiles were simulated for antibody mixtures of 1 to 16348 different antibodies. The correlation (*r*) of simulated AAWS ($\left({\vec{w}}_{\text{sim}}^{i}\right)$) with assigned AAWS ($\vec{h}$) increases with increasing antibody diversity. Both a simulated random peptide library (**X**_sim_) of 255 14-mers as well as assigned AAWS $\vec{h}$ were generated once and kept constant across the whole simulation. Simulated antibody binding profiles were computed with Equation 2, detailed in the results section. Corresponding AAWS were determined with Equation 1. For every mixture of *n*_Ab_-different antibodies, 100 simulations with newly randomly generated antibody mixtures were run. Click here for file

Additional File 6 **Supporting Figure S6: Correlation between 26 physico-chemical properties and the average AAWS of healthy mice**. The average AAWS of healthy mice were correlated with the z-scale published by Sandberg and colleagues [29]. The shown correlation coefficients are Spearman-Rank-correlation coefficients. Same abbreviations were used as by Sandberg and colleagues [29]. MW (molecular weight), TLx (thin layer chromatography at various conditions), vdW (side chain van der Waals volume), NMx (NMR-proton shift at pD = x), logP (10 log (octanol/water) partition coefficient), EHOMO (energy of highest occupied molecular orbital), ELUMO (energy of lowest unoccupied molecular orbital), HOF (heat of formation), POLAR (*α*-polarizability), EN (absolute electronegativity), HA (absolute hardness), Stot (total accessible molecular surface area), Spol (polar accessible molecular surface area), Snp (non-polar accessible molecular surface area), HDONR (number of hydrogen bond donors), HACCR (number of hydrogen bond acceptors), Chpos (indicator of positive charge in side chain), Chneg (indicator of negative charge in side chain). Legend: Red, positive correlation coefficients; blue, negative correlation coefficients. Click here for file
