# Supplementary figures and images for: A minimal model of peptide binding predicts ensemble properties of serum antibodies
Source: BMC Genomics. 2012 Feb 21;13:79. doi: 10.1186/1471-2164-13-79 (PMC3311590; doi:10.1186/1471-2164-13-79)

# Infection of Balb/c mice with *Heligmosomoides bakeri*

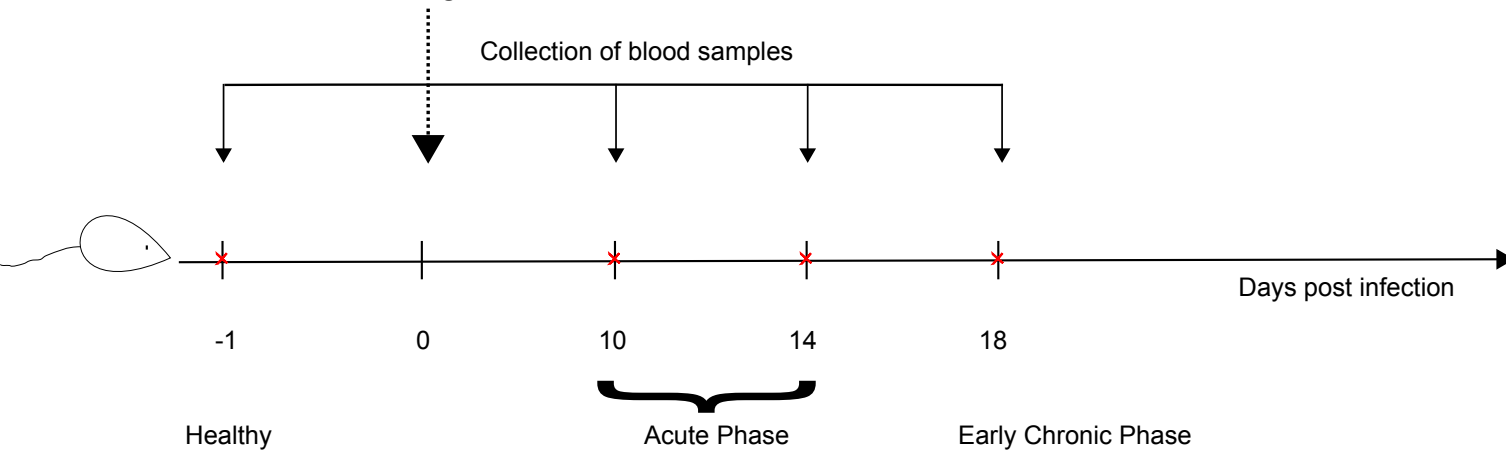

Supplement: Additional File 1 — Supporting Figure S1: Experimental setup: infection of BALB/c mice with Heligmosomoides bakeri and collection of blood samples at three different stages of immune response. Serum samples from 15 BALB/c mice raised under specific pathogen-free conditions were collected. These mice were infected with the intestinal nematode Heligmosomoides bakeri formerly known as Heligmosomoides polygyrus [53]. Further serum samples were collected at 10 dpi (days post infection; 15 samples), at 14 dpi (13 samples), and at 18 dpi (15 samples) totaling 58 serum samples. The serum was isolated and subsequently incubated with random peptide libraries. We categorized the serum samples into healthy (0 dpi; 15 samples), acute phase (10 and 14 dpi; 15 and 13 samples respectively) and early chronic phase (18 dpi; 15 samples), thus delineating the three major stages of immune response of a mouse, before and after primary infection with HB. Practical experimental difficulties reduced the intended number of usable 14 dpi samples from 15 to 13. [file 1471-2164-13-79-S1.PDF]

**A**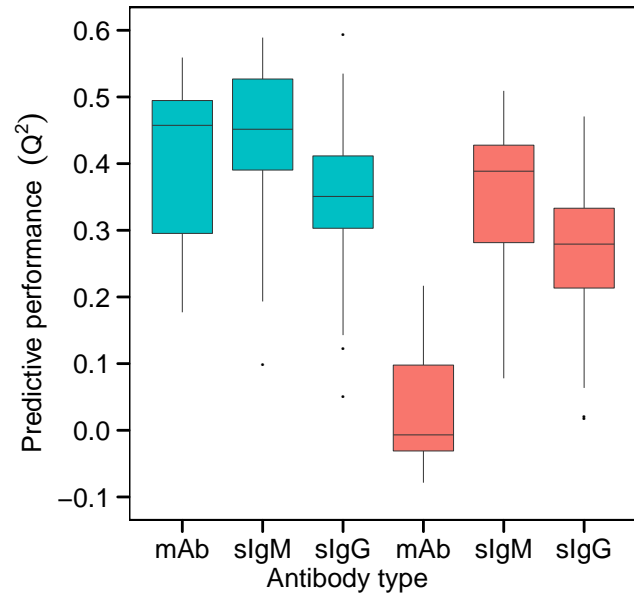**B**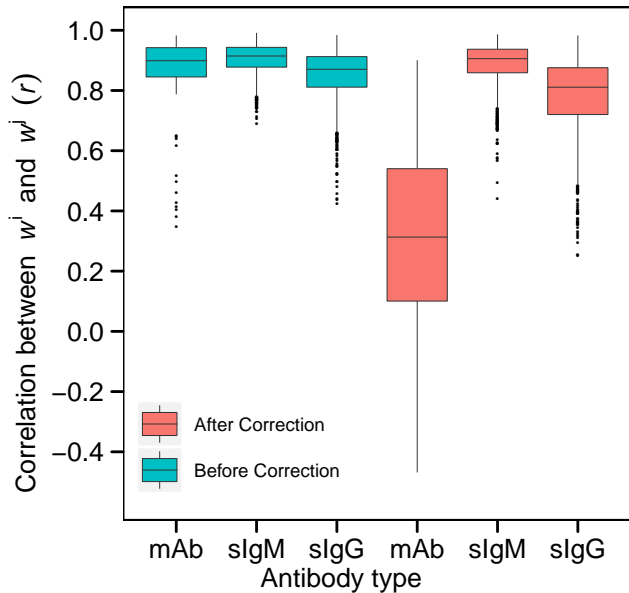

Supplement: Additional File 2 — Supporting Figure S2: Removing the signal of the secondary antibody accentuates differences between binding profiles of monoclonal and serum antibodies. (A) The predictive performance values (Q2) were calculated for monoclonal (mAb) as well as serum IgM (sIgM) and IgG (sIgG) antibody binding profiles before (blue) and after (red) correction of the measured log-transformed signal intensities by removal of the polyclonal secondary antibody-correlated signals using PLS. (B) Shown is the pairwise correlation (r) of the corresponding AAWS w →j. For the two statistical measures, signal correction entails a significant decrease in the mAb median, whereas sIgM and sIgG medians remain largely unchanged. Both before and after secondary antibody correction of antibody binding profiles, sIgM profiles have higher predictive performance (Q2) and a higher median pairwise correlation (r) of AAWS than sIgG profiles. In (A) and (B), mAb signifies antibody binding profiles from 13 monoclonal antibodies and sIgM/sIgG serum IgM and serum IgG binding profiles from 58 BALB/c mice sera, respectively. Antibody binding profiles were measured with the standard peptide library of 255 14-mers. Corresponding AAWS (w →j) were determined with Equation 1. [file 1471-2164-13-79-S2.PDF]

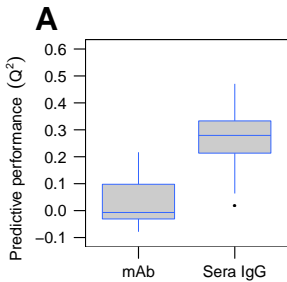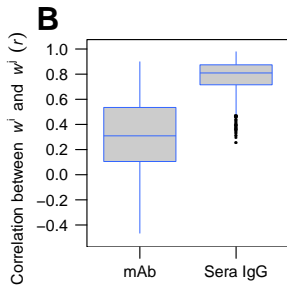

Supplement: Additional File 3 — Supporting Figure S3: Predictive performance and pairwise correlation of amino acid-associated weights are higher for serum IgG than for monoclonal antibodies. (A) Predictive performance values (Q2) were calculated for monoclonal (mAb) and serum IgG antibody (Sera IgG) binding profiles. (B) Shown is the pairwise correlation (r) of the corresponding AAWS w →j. In both (A) and (B) mAb signifies antibody binding profiles from 13 monoclonal antibodies and Sera IgG binding profiles from 58 BALB/c mice sera. Differences in predictive performance (Q2) and pairwise correlation (r) of AAWS between monoclonal and serum IgG antibodies are significant (p < 0.001). Antibody binding profiles were measured with the standard peptide library of 255 14-mers. Corresponding AAWS (w →j) were determined with Equation 1. [file 1471-2164-13-79-S3.PDF]

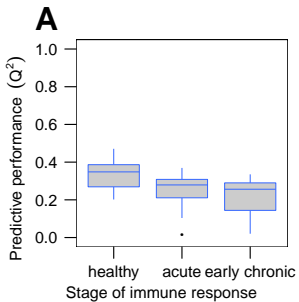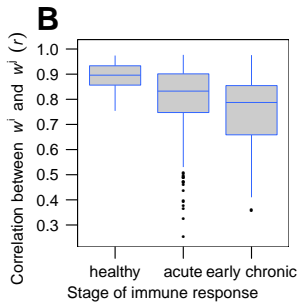

Supplement: Additional File 4 — Supporting Figure S4: Predictive performance and pairwise correlation of amino acid-associated weights decrease for serum IgG antibodies during the course of the immune response. (A) Predictive performance values (Q2) were computed from serum IgG antibody binding profiles across three stages of immune response: healthy, acute, early chronic. (B) Shown is the pairwise correlation (r) of the corresponding AAWS w →simj. Number of BALB/c mice serum samples: 15 from healthy mice, after infection with HB: 15 samples taken at 10 dpi and 13 samples taken at 14 dpi (acute phase) and 15 samples taken at 18 dpi (early chronic) totaling 58 BALB/c serum samples. Differences in predictive performance (Q2) between both healthy and early chronic phase mice and healthy and acute phase mice are significant (p < 0.05) as are differences in pairwise correlation (r) between all three stages of immune response (p < 0.001). Antibody binding profiles were measured with the standard peptide library of 255 14-mers. Corresponding AAWS (w →j) were computed using Equation 1. [file 1471-2164-13-79-S4.PDF]

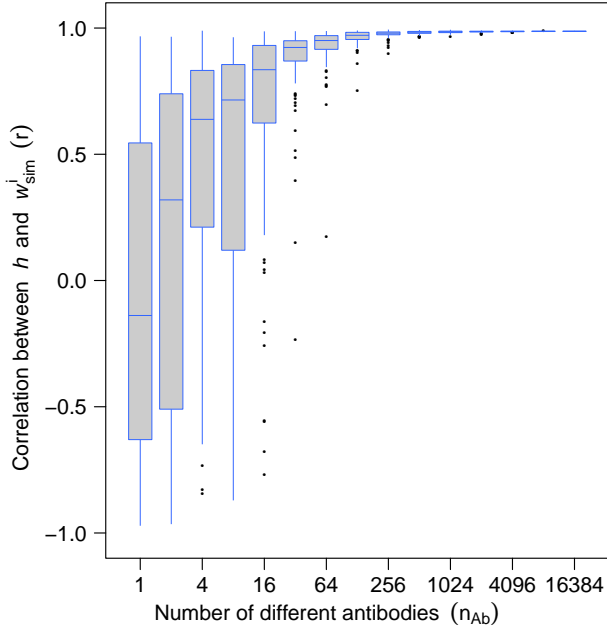

Supplement: Additional File 5 — Supporting Figure S5: Simulations show that recovery of assigned amino acid-associated weights is positively correlated to antibody diversity. This Figure is complementary to Figure 3. Antibody binding profiles were simulated for antibody mixtures of 1 to 16348 different antibodies. The correlation (r) of simulated AAWS ((w →simi)) with assigned AAWS (h →) increases with increasing antibody diversity. Both a simulated random peptide library (Xsim) of 255 14-mers as well as assigned AAWS h → were generated once and kept constant across the whole simulation. Simulated antibody binding profiles were computed with Equation 2, detailed in the results section. Corresponding AAWS were determined with Equation 1. For every mixture of nAb-different antibodies, 100 simulations with newly randomly generated antibody mixtures were run. [file 1471-2164-13-79-S5.PDF]

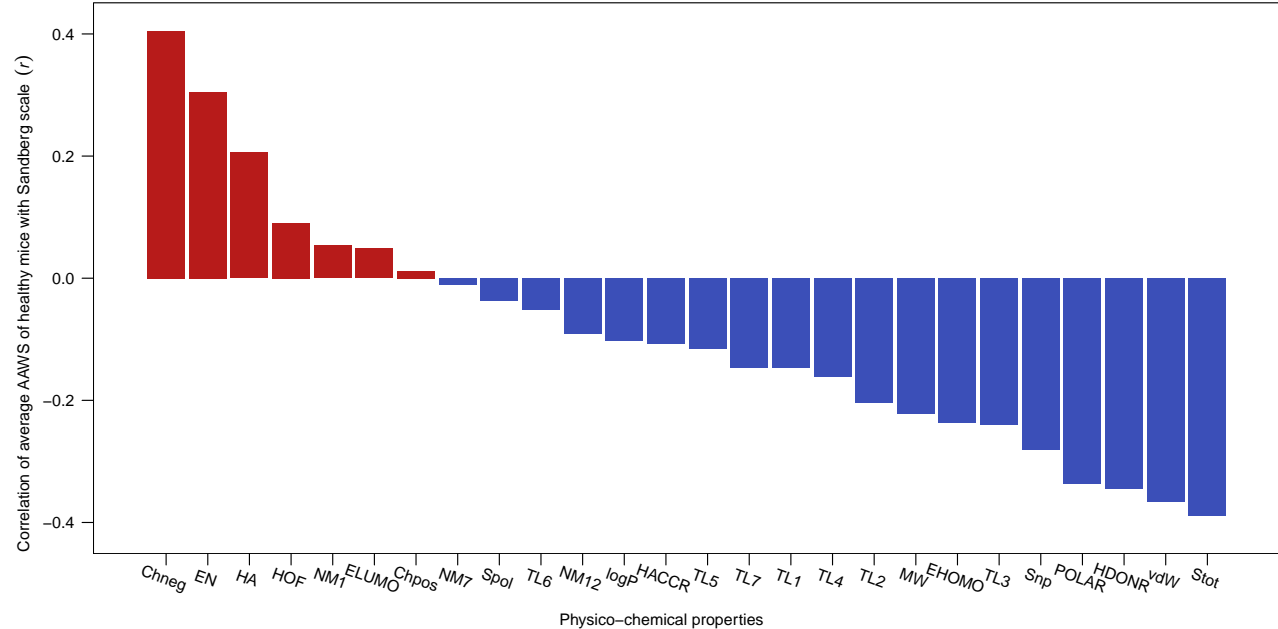

Supplement: Additional File 6 — Supporting Figure S6: Correlation between 26 physico-chemical properties and the average AAWS of healthy mice. The average AAWS of healthy mice were correlated with the z-scale published by Sandberg and colleagues [29]. The shown correlation coefficients are Spearman-Rank-correlation coefficients. Same abbreviations were used as by Sandberg and colleagues [29]. MW (molecular weight), TLx (thin layer chromatography at various conditions), vdW (side chain van der Waals volume), NMx (NMR-proton shift at pD = x), logP (10 log (octanol/water) partition coefficient), EHOMO (energy of highest occupied molecular orbital), ELUMO (energy of lowest unoccupied molecular orbital), HOF (heat of formation), POLAR (α-polarizability), EN (absolute electronegativity), HA (absolute hardness), Stot (total accessible molecular surface area), Spol (polar accessible molecular surface area), Snp (non-polar accessible molecular surface area), HDONR (number of hydrogen bond donors), HACCR (number of hydrogen bond acceptors), Chpos (indicator of positive charge in side chain), Chneg (indicator of negative charge in side chain). Legend: Red, positive correlation coefficients; blue, negative correlation coefficients. [file 1471-2164-13-79-S6.PDF]
